# Effect of Annealing on Microstructure and Tensile Behavior of CoCrNi Medium Entropy Alloy Processed by High-Pressure Torsion

**DOI:** 10.3390/e20110849

**Published:** 2018-11-06

**Authors:** Praveen Sathiyamoorthi, Jae Wung Bae, Peyman Asghari-Rad, Jeong Min Park, Jung Gi Kim, Hyoung Seop Kim

**Affiliations:** 1 Department of Material Science and Engineering, Pohang University of Science and Technology (POSTECH), Pohang 37673, Korea; 2 Center for High Entropy Alloys, Pohang University of Science and Technology (POSTECH), Pohang 37673, Korea; 3 Graduate Institute of Ferrous Technology (GIFT), Pohang University of Science and Technology (POSTECH), Pohang 37673, Korea

**Keywords:** medium entropy alloy, high-pressure torsion, partial recrystallization, tensile strength

## Abstract

Annealing of severely plastic deformed materials is expected to produce a good combination of strength and ductility, which has been widely demonstrated in conventional materials. In the present study, high-pressure torsion processed CoCrNi medium entropy alloy consisting of a single face-centered cubic (FCC) phase with a grain size of ~50 nm was subjected to different annealing conditions, and its effect on microstructure and mechanical behavior was investigated. The annealing of high-pressure torsion processed CoCrNi alloy exhibits partial recrystallization and near full recrystallization based on the annealing temperature and time. The samples annealed at 700 °C for 2 min exhibit very fine grain size, a high fraction of low angle grain boundaries, and high kernel average misorientation value, indicating partially recrystallized microstructure. The samples annealed for a longer duration (>2 min) exhibit relatively larger grain size, a low fraction of low angle grain boundaries, and low kernel average misorientation value, indicating nearly full recrystallized microstructure. The annealed samples with different microstructures significantly influence the uniform elongation, tensile strength, and work hardening rate. The sample annealed at 700 °C for 15 min exhibits a remarkable combination of tensile strength (~1090 MPa) and strain to failure (~41%).

## 1. Introduction

High entropy alloys (HEAs), also known as compositionally complex alloys, baseless alloys, and concentrated solid solution alloys, exhibit unique and remarkable properties as compared with the conventional alloys [1,2,3]. The unique properties of HEAs are mainly attributed to the distinct alloy design concept based on multi-principal elements [4,5]. The alloys with multi-principal elements are widely classified into medium entropy alloys (MEAs) and HEAs based on the configurational entropy [5,6]. Among the HEAs, alloys based on the Co–Cr–Fe–Mn–Ni system, Al–Co–Cr–Cu–Fe–Ni system, and refractory elements are widely reported in the literature [1,2,5].

Recently, CoCrNi MEA has received widespread attention from researchers because of its unique properties such as exceptional fracture toughness at cryogenic temperature, high friction stress, superior dynamic shear properties, and annealing-induced hardening at intermediate temperatures (500–600 °C) [7,8,9,10]. It is widely reported that HEAs with a single face-centered cubic (FCC) phase exhibit low yield strength [11,12,13]. Several strategies have been reported to increase the strength of a single FCC phase HEAs by solid solution strengthening, precipitation strengthening, grain boundary strengthening, and partial recrystallization through thermo-mechanical treatments [11,14,15,16]. Among the several strategies to enhance the mechanical properties, thermo-mechanical treatment is an effective technique to achieve a good combination of strength and ductility by achieving microstructure with different grain size and/or different volume fraction of secondary phases [11,17,18]. The CoCrNi MEA with a single FCC phase, like any other HEA with a single FCC phase, also exhibits lower yield strength [14,19]. Recently, it has been reported that the addition of 3 at% W resulted in a 20% increase in intrinsic strength [14]. In another study, cold rolling followed by annealing of CoCrNi alloy is reported to have an ultra-fine-grained size (~750 nm) with a good combination of tensile strength (964 MPa) and strain to failure (66%) [20]. In this connection, high-pressure torsion (HPT) followed by annealing has been effectively used in conventional alloys and HEAs to improve their mechanical properties [21,22,23]. Generally, HPT is an effective process in fabricating materials with nano-grains or ultra-fine grained structure by simultaneous application of torsion and high hydrostatic pressure [22]. The HPT processed CoCrNi MEA is reported to have a grain size of ~50 nm with an exceptional ultimate tensile strength of ~2.2 GPa and a considerable strain to failure of 9% [24]. It is also reported that annealing of the HPT processed CoCrNi MEA in the temperature range of 500–600 °C has resulted in an increase in hardness due to a decrease in dislocation density and grain boundary relaxation [7]. However, the systematic investigation of post HPT annealing of CoCrNi MEA with respect to microstructure evolution and strength enhancement is limited. 

Thus, the present study is aimed at achieving a better combination of strength and ductility in the HPT processed CoCrNi MEA by post-HPT annealing. Consequently, in the present study, the post-HPT annealing effect on the microstructural and mechanical behaviors of CoCrNi MEA is investigated. 

## 2. Experimental Methods

The CoCrNi alloy for the present study was fabricated using a vacuum induction melting of pure metals followed by homogenization, cold rolling, recrystallization, HPT, and annealing of HPT samples. The detailed procedure about the fabrication and secondary processing route can be found in our earlier publications [7,24]. The microstructure of starting material for HPT consists of a single FCC phase with an average grain size of ~19 µm and profuse annealing twins [24]. After the HPT process, the microstructure consists of a very fine grain size of ~50 nm with a high density of nano-twins and stacking faults [24]. In this study, the HPT processed sample with a single FCC phase was subjected to different heat treatment conditions in the Ar atmosphere to investigate the effect of post-HPT annealing. The post-HPT annealed samples were characterized using X-ray diffraction (XRD, Rigaku D/MAX-2500), field emission scanning electron microscopy (FE-SEM, FEI XL30S) coupled with electron backscatter diffraction (EBSD), Vickers hardness, and tensile testing equipment (Instron 1361). The deformed microstructures of post tensile samples were characterized using a focused ion beam (FIB) coupled with transmission Kikuchi diffraction analysis (TKD, FEI-FIB/TKD).

Tensile samples (dimension: 1.5 mm gauge length and 1 mm gauge width) were cut from the annealed HPT disc using electric discharge machining. Tensile tests were carried out at a quasi-static strain rate of 10^−3^ s^−1^ and the strain on the sample was measured using digital image correlation (DIC, ARAMIS v6.1, GOM Optical Measuring Techniques). The Vickers hardness measurement was performed using a load of 300 gf and a dwell time of 15 s. For EBSD experiment, the samples were mechanically polished using SiC carbide papers followed by diamond and colloidal polishing. For TKD experiment, a transparent sample near to the fracture surface of deformed tensile samples was lifted out by FIB.

## 3. Results and Discussion

**Figure 1:**
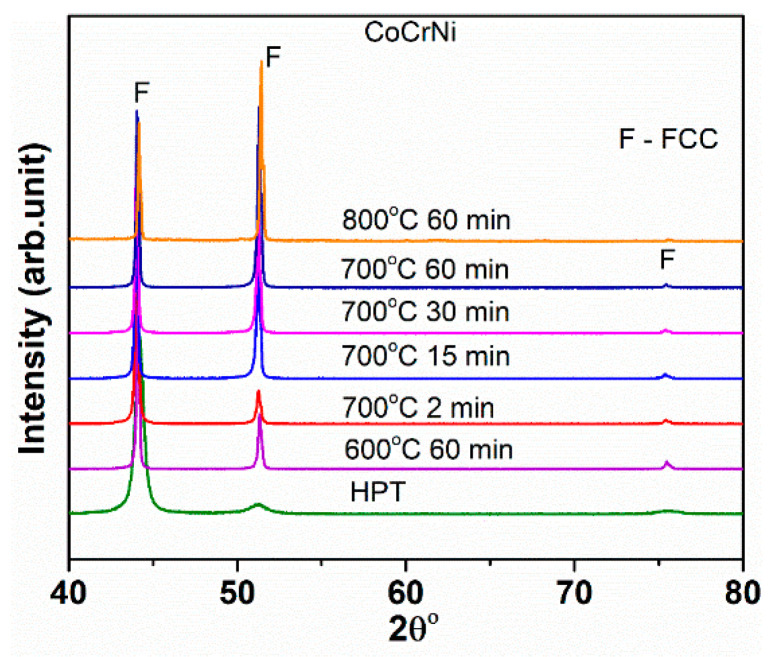
X-ray diffraction (XRD) patterns of CoCrNi alloy in different processing conditions illustrating the presence of a single face-centered cubic (FCC) phase.

Figure 1 shows the XRD patterns of CoCrNi alloy under different processing conditions: HPT and post-HPT annealing. It can be seen that in all the processing conditions, the alloy exhibits a single FCC phase. 

The formation of sigma phase/secondary phases is widely reported for HEAs processed by HPT and annealing. In Al_0.5_CoCrFeMnNi HEA, the formation of sigma phase is reported when the HPT processed sample is annealed at 800 °C for 1 h [25]. Similarly, for the HPT processed CrFe_2_NiMnV_0.25_C_0.125_ HEA, sigma phase has been observed during annealing at 550 °C [26]. In CoCrFeMnNi HEA, the formation of sigma and secondary nano-phases have been reported after annealing of the HPT processed sample [27]. However, in the present alloy, there is no evidence of formation of sigma phase or secondary phase in the temperature range of 600–800 °C. The formation of sigma phase in this alloy was also not observed when annealed in the temperature range of 500–600 °C [7].

**Figure 2:**
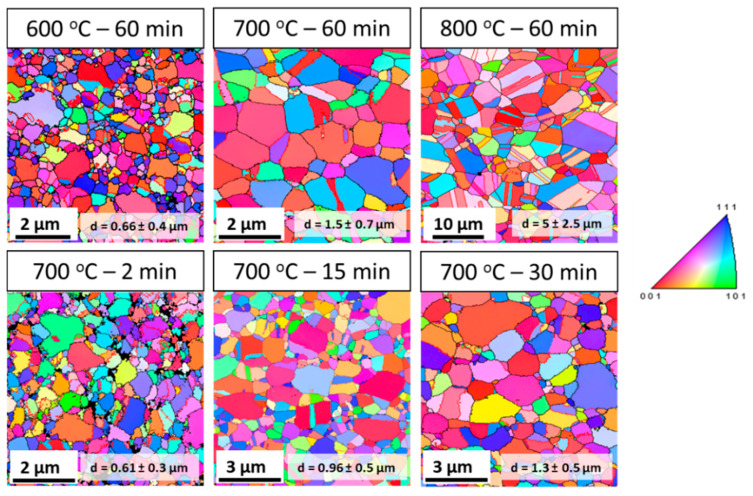
Electron backscatter diffraction (EBSD) inverse pole figure map of post-high-pressure torsion (HPT) annealing samples. The color scale on the right side corresponds to [001] inverse pole figure.

**Figure 3:**
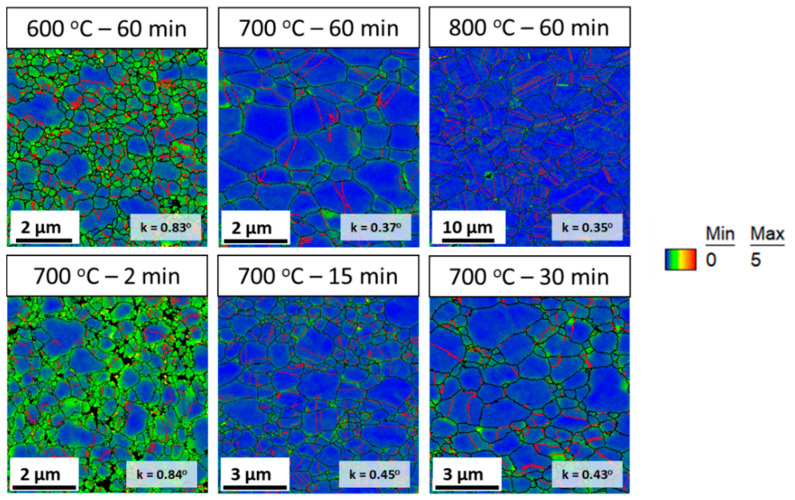
Kernel average misorientation (KAM) map of post-HPT annealing samples, and the corresponding color scale (right side).

**Figure 4:**
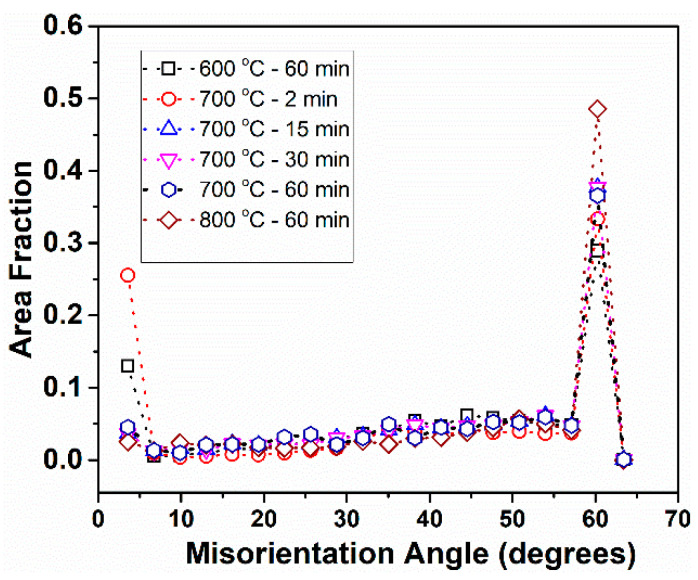
Misorientation angle chart of HPT processed CoCrNi alloy after annealing at different temperatures and times.

Figure 2 shows the inverse pole figure (IPF) map of the post-HPT annealed samples, and the corresponding kernel average misorientation (KAM) map and misorientation angle chart are shown in Figure 3 and Figure 4, respectively. 

The IPF map indicates an increase in grain size with increasing annealing temperature and time. The samples annealed at 600 °C for 60 min and 700 °C for 2 min consist of a microstructure with almost similar average grain size (~600–670 nm), while the samples annealed at 700 °C over 2 min and 800 °C for 60 min show that the average grain size is in the range of approximately 960 nm to 5 µm.

The KAM map is generally used to represent the strain distribution based on the dislocation density. The KAM maps in Figure 3 were estimated up to the third nearest neighbor kernel with a maximum misorientation of 5°. The KAM map (Figure 3) shows that the strain distribution in samples annealed at 600 °C for 60 min and 700 °C for 2 min is relatively high as compared with samples at other annealing conditions. This clearly indicates that the strain induced into the material by HPT processing is not completely recovered in samples annealed at 600 °C for 60 min and 700 °C for 2 min. In general, deformed samples exhibit a larger KAM value (>1°), while recrystallized samples exhibit a lower KAM value (<1°) [28]. An almost similar average KAM value of approximately 0.83–0.84 is observed for samples annealed at 600 °C for 60 min and 700 °C for 2 min, while the average KAM value is in the range of 0.37°–0.45° for samples annealed at 700 °C over 2 min and 800 °C for 60 min.

The misorientation chart (Figure 4) indicates that the fraction of low angle grain boundaries is higher in samples annealed at 600 °C for 60 min and 700 °C for 2 min, but decreases significantly with increasing annealing time and temperature. However, a high fraction of 60° misorientation angle can be seen in all the conditions, with the fraction increasing with temperature and time. The presence of a high fraction of low angle grain boundaries indicates that the samples are not fully recrystallized, and the high fraction of 60° misorientation angle indicates the presence of abundant annealing twins.

Based on the IPF map, KAM map, and misorientation angle chart, the annealed samples can be divided into two categories: (a) microstructure with fine grain size, high KAM value, and high fraction of low angle grain boundaries (partially recrystallized); and (b) microstructure with relatively coarser grain size, low KAM value, and low fraction of low angle grain boundaries (nearly fully recrystallized). Accordingly, the sample annealed at 600 °C for 60 min, and 700 °C for 2 min will be referred to as samples with partially recrystallized microstructures. Similarly, the samples annealed at 700 °C for 15 min, 700 °C for 30 min, 700 °C for 60 min, and 800 °C for 60 min will be referred to as samples with recrystallized microstructures. 

**Figure 5:**
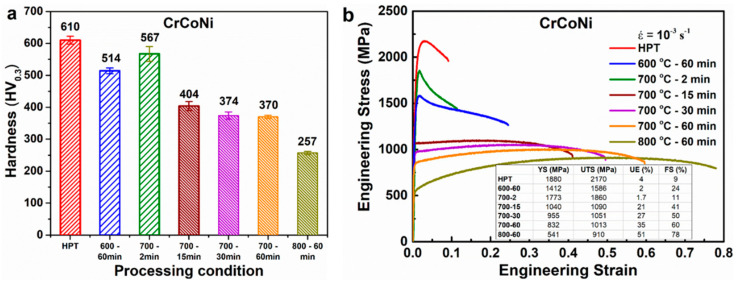
Mechanical properties of CoCrNi alloy in different processing conditions. (**a**) Hardness and (**b**) engineering stress–strain curve.

The mechanical properties of CoCrNi alloy at different processing conditions were assessed by Vickers hardness measurement and uniaxial tensile test. The hardness values under different processing conditions are shown in Figure 5a. The hardness of the HPT processed sample decreases with increasing annealing temperature and time. The annealed HPT samples with partially recrystallized microstructure show the smallest decrease in hardness as compared with the samples with near fully recrystallized microstructure. This can be attributed to the fine grain size and high KAM value in the samples with partially recrystallized microstructure. 

The engineering stress–strain curves of CoCrNi alloy at different processing conditions are shown in Figure 5b. The tensile curves clearly indicate that the annealing of the HPT processed sample leads to a reasonably good combination of strength and ductility. The tensile strength of the annealed samples follows a similar trend to that of hardness value. The annealed samples with partially recrystallized microstructure show the lowest yield strength reduction compared with the samples with recrystallized microstructure. In steels, it has been reported that the strength increases with an increase in pre-strain [29]. As KAM indicates the strain distribution based on the dislocation density, the annealed samples with high KAM can be regarded as a material with pre-strain. Thus, high strength in the samples with partially recrystallized microstructure can be attributed to the fine grain size and high KAM value.

The post-HPT annealing has significantly improved the uniform elongation and strain to fracture at the cost of a reduction in strength for samples with recrystallized microstructure. It is interesting to note that only the strain to fracture has been improved significantly and not the uniform elongation for the samples with partially recrystallized microstructure. The uniform elongations in the samples annealed at 600 °C for 60 min and 700 °C for 2 min are almost similar to each other and are also similar to the HPT processed sample. However, the strain to fracture is significantly higher in the samples annealed at 600 °C for 60 min and 700 °C for 2 min than the HPT processed sample, with the sample annealed at 600 °C for 60 min showing higher elongation to fracture. 

In high-grade pipeline steels, it has been reported that the samples with a high percentage of low angle grain boundaries resulted in the smaller difference between yield strength and tensile strength [30]. In the present study, the samples with a high fraction of low angle grain boundaries exhibit a smaller difference between the yield strength and tensile strength. In addition, it has been shown that defects such as dislocation and vacancies can pass through the low angle grain boundaries, unlike the pile-up of defects at high angle grain boundaries [31]. This could be a possible reason for reduced work hardenability, thereby resulting in reduced uniform elongation in materials with a high fraction of low-angle grain boundaries. In addition, low angle grain boundaries are considered to be resistant to intergranular fracture in metallic materials [32]. This could be a possible reason for reasonable post-necking deformation in samples with partially recrystallized microstructure. Thus, the presence of low angle grain boundaries resulted in an increase in strain to fracture without enhancement in uniform elongation. 

In conventional alloys, enhancement of uniform elongation in post-deformed annealed samples is observed when the fraction of recrystallized grains exceeds a certain volume fraction. In Cu–Al alloys, a reasonable improvement in uniform elongation is observed when the recrystallized grains are over 80% of the volume fraction [33]. In nanostructured Cu processed by rolling at liquid nitrogen, an increase in uniform elongation is reported when the fraction of secondary recrystallized grains is about 25% [34]. In the present study, an increase in uniform elongation is observed when the fraction of low-angle grain boundaries and the average KAM value is lower than 5% and 0.5°, respectively. 

The shape of the flow curves (Figure 5) is distinct between the samples with partially recrystallized and recrystallized microstructures, clearly indicating the influence of microstructure on the flow curve. In samples with the partially recrystallized microstructure, the flow curve shows softening behavior. The rate of softening in the sample annealed at 600 °C for 60 min is different from that of the sample annealed at 700 °C for 2 min, with the one at 700 °C for 2 min showing pronounced softening. As the average KAM value and the average grain size are almost similar for 700 °C for 2 min and 600 °C for 60 min samples, the pronounced softening in the one at 700 °C for 2 min clearly indicates that the fraction of low angle grain boundaries significantly influences the softening behavior. Thus, the sample annealed at 700 °C for 2 min with a higher fraction of low-angle grain boundaries shows more pronounced softening than the 600 °C for 2 min sample. In the samples with recrystallized microstructure, the flow curves show strain hardening behavior, and the strain hardening ability is observed to increase with increasing annealing temperature and time. 

**Figure 6:**
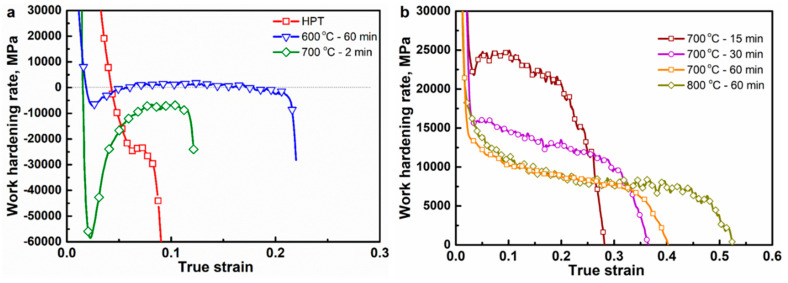
Work hardening rate as a function of true strain for samples under different processing conditions.

In order to further understand the flow curve behavior, the work hardening rate (WHR) curves are plotted as a function of true strain for different processing conditions and are shown in Figure 6. In general, the WHR is not discussed with respect to plastic deformation after the plastic instability. However, the WHR after plastic instability is also shown in Figure 6a to understand the enhancement in strain to fracture without a significant enhancement of uniform elongation in samples with partial recrystallization. For the HPT sample, the WHR decreases rapidly and enters into the softening after yielding, and decreases continuously till fracture. This is commonly observed in severely deformed or nano-grained materials because of the limited dislocation activity and ineffectiveness in dislocation generation and accumulation during deformation. For the samples with a partially recrystallized microstructure, the WHR decreases initially and enters the softening region, and with further strain, the WHR curve increases and slowly approaches a peak, and then decreases gradually. The amount of increase in WHR from softening is higher for the sample annealed at 700 °C for 2 min than that at 600 °C for 60 min. Interestingly, the WHR curve of the 600 °C for 60 min sample increases after softening and reaches hardening region (i.e., from a negative value to positive value of WHR) and then decreases again as the strain approaches the fracture strain. As previously mentioned, the difference softening behavior in the partially recrystallized microstructure samples can be attributed to the difference in the fraction of low angle grain boundaries. The increase in WHR curve in the softening zone can be a possible reason for the increase in strain to failure without an increase in uniform elongation in the annealed samples with partially recrystallized microstructure. 

The WHR curves of samples with recrystallized microstructure are shown in Figure 6b. The WHR curves show three distinct stages of WHR. In the first stage (Stage A), the WHR decreases rapidly at the initial stage of plastic deformation (elastic to plastic), and is observed in all the annealed samples. In 700 °C for 60 min and 800 °C for 60 min samples, the second stage (Stage B) shows a gradual decrease in WHR followed by a slow decrease (Stage C) with the increase in true strain. In the 700 °C for 30 min sample, the second stage of WHR is very narrow, and is not significant as in 700 °C for 60 min and 800 °C for 60 min samples. In the 700 °C for 15 min annealed sample, Stage B shows an increase in WHR followed by the slow decrease in WHR (Stage C).

The difference in WHR stage, especially Stage B, of the annealed samples can be attributed to the difference in grain size and the amount of strain present in the sample before tensile testing [15,35]. The increase in WHR in Stage B has been reported for ultra-fine grained CoCrMnFeNi HEAs, and it is attributed to the yield drop phenomenon observed in ultra-fine grained materials [15]. In commercial pure Ti, it is demonstrated that the increase in WHR in Stage B is observed when the pre-strain reaches 3.5% and is attributed to the presence of high density of dislocations and deformation twinning in pre-strained samples. In the present study, the increase in WHR in Stage B of the 700 °C for 15 min sample could be attributed to the fine grain size (~960 nm) and the presence of strain (KAM value ~0.45°) (Figure 3). As the grain size and KAM value of the 700 °C for 30 min samples fall in between the those of the 700 °C for 15 min sample and 700 °C for 60 min sample, Stage B is observed only for a narrow strain as there could be a balance between increase and gradual decrease in WHR. 

**Figure 7:**
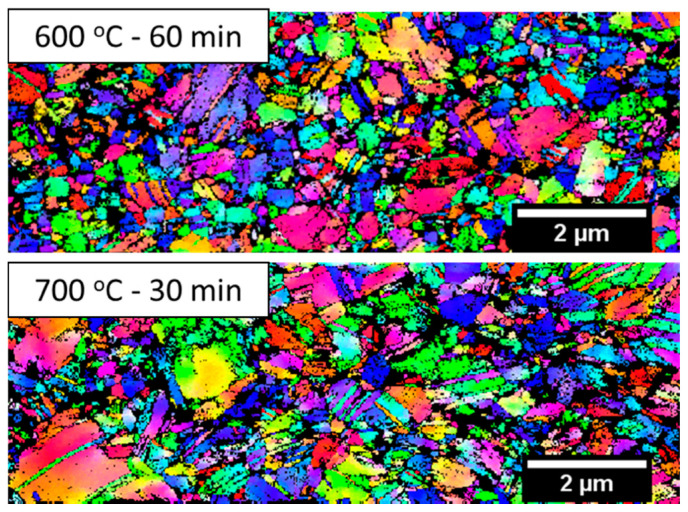
Transmission Kikuchi diffraction inverse pole figure map of post-deformed samples of 600 °C for 60 min and 700 °C for 30 min annealed samples.

Figure 7 shows the deformed microstructure (transmission Kikuchi diffraction inverse pole figure map) near the fracture surfaces of the samples with partially recrystallized microstructure (600 °C for 60 min) and recrystallized microstructure (700 °C for 30 min). As a result of the large strain developed in the material, the confidence index is low, and there are some unindexed regions in Figure 7. Both the microstructures show the presence of fine annealing twins. It is to be noted that the formation of deformation twins is difficult to form in ultra-fine grained materials as the critical twining stress increases with decreasing grain size [36,37]. Indeed, the formation of deformation twinning is not observed in ultra-fine grained CoCrMnFeNi HEA [15]. The presence of very fine twins in Figure 7 could be annealing twins, and the deformation twins may not have formed as a result of the fine grain size of the annealed samples in the present study. Besides, the WHR curve also did not show a plateau region indicating the formation of twins during deformation. 

**Figure 8:**
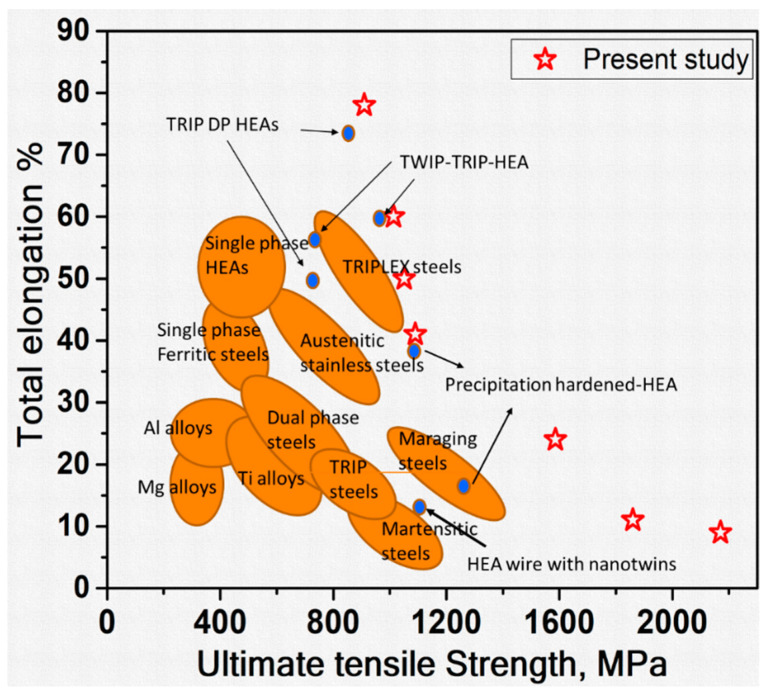
Comparison of tensile properties of HPT processed and post-HPT annealed CoCrNi alloy with other high strength high entropy alloys (HEAs) and conventional alloys (This figure is adopted and modified from the literature [38]. The values for HEA wire with nano-twins are taken from the literature [39]).

Figure 8 shows the comparison of tensile properties of the present study with the conventional alloys and other high strength HEAs. (Figure 8 is adopted and modified from the literature [38]. The values for HEA wire with nano-twins are taken from the literature [39]). It is clear that the strengths of the CoCrNi in HPT processed state and after annealing conditions are quite high as compared with the other HEAs and other high strength conventional alloys.

The superior mechanical properties achieved in the present alloy can be primarily attributed to the presence of fine grains, the formation of fine annealing twins, high shear stress, and high friction stress. It is well known that the strength increases with the decrease in grain size based on the Hall–Petch relation [9,40]. The presence of very fine twins can enhance the mechanical properties by reducing the dislocation mean free path and resisting the dislocation slip, a mechanism very similar to the Hall–Petch relation [33]. The CoCrNi alloy is reported to have very low stacking fault energy and high shear stress [19,41]. In materials with high shear stress and low stacking fault energy, the cross-slip during deformation becomes difficult and thereby enhances the mechanical properties [36,42]. It is reported that the friction stress of CoCrNi alloy is higher than that of many conventional FCC metals because of the fluctuation of Peierls potential for dislocation motion, and it can enhance the strength of the material [9]. Thus, the presence of fine grains, fine annealing twins, high shear stress, high friction stress, and low-angle grain boundaries resulted in superior mechanical properties in the post-HPT annealed CoCrNi samples.

## 4. Summary

In the present study, the effect of annealing on the microstructural and mechanical properties of HPT processed CoCrNi HEA was investigated. The results clearly indicate that the initial microstructure plays an important role in the mechanical properties of CoCrNi alloy. The initial microstructure with fine grain size of ~660 nm, high fraction of low angle grain boundaries, and high KAM value shows strain softening followed by strain hardening, whereas only strain hardening is observed for the microstructure with grain size >900 nm, a low fraction of low-angle grain boundaries, and low KAM value. The sample annealed at 700 °C for 15 min shows the optimized room temperature mechanical properties with a remarkable combination of strength (1090 MPa) and strain to failure (41%). Thus, the present study demonstrates that the severe plastic deformation followed by annealing is one of the effective ways of enhancing the mechanical properties in CoCrNi MEA.
